# Metabolites from nematophagous fungi and nematicidal natural products from fungi as alternatives for biological control. Part II: metabolites from nematophagous basidiomycetes and non-nematophagous fungi

**DOI:** 10.1007/s00253-015-7234-5

**Published:** 2016-01-04

**Authors:** Thomas Degenkolb, Andreas Vilcinskas

**Affiliations:** Institute for Insect Biotechnology, Justus-Liebig-University of Giessen, Heinrich-Buff-Ring 26-32, D-35392 Giessen, Germany; Department of Bioresources, Fraunhofer Institute for Molecular Biology and Applied Ecology, Winchester Strasse 2, D-35394 Giessen, Germany

**Keywords:** Phytoparasitic nematodes, Nematicides, Nematophagous fungi, Secondary metabolites, Biocontrol

## Abstract

In this second section of a two-part mini-review article, we introduce 101 further nematicidal and non-nematicidal secondary metabolites biosynthesized by nematophagous basidiomycetes or non-nematophagous ascomycetes and basidiomycetes. Several of these compounds have promising nematicidal activity and deserve further and more detailed analysis. Thermolides A and B, omphalotins, ophiobolins, bursaphelocides A and B, illinitone A, pseudohalonectrins A and B, dichomitin B, and caryopsomycins A–C are excellent candidates or lead compounds for the development of biocontrol strategies for phytopathogenic nematodes. Paraherquamides, clonostachydiol, and nafuredins offer promising leads for the development of formulations against the intestinal nematodes of ruminants.

## Introduction

### Metabolites from nematophagous basidiomycetes

#### General remarks

The chemical ecology of nematophagous fungi is still far from understood. Little has been done to screen for metabolites in nematophagous fungi, or nematicidal metabolites in other fungi, since the pioneering studies by Stadler and colleagues published in the 1990s (Stadler et al. 1993a, b, 1994a, b, c, d). In the first part of this review, we discussed 83 primary and secondary metabolites from nematophagous ascomycetes (Degenkolb and Vilcinskas, in press). In this second installment, we consider nematicidal metabolites from nematophagous basidiomycetes and from those fungi that are currently regarded as non-nematophagous species. The numbering system for the compounds introduced here begins at **84** to provide continuity with the first part of the review.

Given that species parasitizing nematodes or their eggs are found in all major fungal phyla including *Chytridiomycota*, *Ascomycota*, *Basidiomycota*, and also the *Zoopagomycotina* and *Mucormycotina*,^1^ multiple and independent evolution of nematophagy was hypothesized (Barron 1977). The scenario of nematode-fungus associations may be far more complex than previously thought. This was recently exemplified by Morris and Hajek (2014) who reported on the parasitic nematode *Deladenus siricidicola* (*Tylenchida*: *Neotylenchidae*), which is used for biocontrol of the invasive pine-killing woodwasp *Sirex noctilio* (*Hymenoptera*: *Siricidae*). In its mycophagous phase, *D. siricidicola* feeds exclusively on the growing hyphal tips of its basidiomycete host *Amylostereum areolatum* (*Russulales*: *Amylostereaceae*). The presence of woodwasp larvae triggers the nematode to change its life style—it invades the wasp larvae and sterilizes most of them. Notably, the white-rot fungus, which has so far been thought to serve as food source for *Deladenus* sp., was also shown to (1) invade the vulva of adult female mycophagous nematodes and (2) to kill and invade nematode eggs. Eggs were parasitized by the hyphal tips of the fungus whereas cystidia seemed to colonize the vulva of adults. It remains to be clarified if a toxin is also involved in the infection process,

Authors are aware of the fact that missing evidence does not necessarily imply a non-nematophagous life style of a fungus. However, for reasons of convenience and consistency with literature, we prefer to retain the terminus “non-nematophagous” for those associations without evidence for nematophagy.

#### Metabolites from the genus *Pleurotus*

The small but monophyletic family *Pleurotaceae* comprises nematophagous white-rot fungi (Thorn et al. 2000; Kirk et al. 2008). Members of the genus *Pleurotus*, such as the oyster mushroom *Pleurotus ostreatus*, have been shown to secrete tiny toxin-containing droplets, which effectively paralyze a nematode without killing it within 30 s of contact. The prey is subsequently penetrated by the fungal trophic hyphae and digested within 24 h (Thorn and Barron 1984; Barron and Thorn 1987).

The first nematicidal compound isolated from the genus *Pleurotus* was (*E*)-2-decenedioic acid (**84**). *P. ostreatus* NRRL 3526 (= ATCC 90520) was grown for 30 days at room temperature (21–23 °C) on autoclaved, damp wheat straw. Thereafter, an aqueous extract of the colonized substrate was filtered, and the filtrate was freeze-dried. After reconstitution of the lyophilizate in water, the organic fraction of the extract was further purified, finally by HPLC of the acetone-soluble fraction. The nematicidal principle, compound **84**, which eluted as a single peak, was characterized by MS and NMR. An aqueous solution of pure **84** at a concentration of 300 μg/ml caused the immobilization of 95 % of a test population of the nematode *Panagrellus redivivus* within 1 h. Notably, this effect could not be reversed by rinsing the treated nematodes with deionized water. Organic extracts of a static straw culture have not been prepared and investigated for possible nematicidal activity (Kwok et al. 1992).

Six further nematicidal compounds (**1**, **85**–**89**) were isolated from an 11-day fermentation of the pale oyster *Pleurotus pulmonarius*. All of the compounds were found in the mycelial extracts, whereas the culture broth only contained compounds **86–89**. Compound **85** was (*S*)-(9*Z*,11*E*)-13-hydroxy-9,11-octadecadienoic acid (also known as *S*-coriolic acid), and this along with compound **1** (linoleic acid) exhibited the most potent nematicidal activity. The median lethal concentrations (LC_50_) against *Caenorhabditis elegans* were less pronounced for *p*-anisaldehyde (**86**), *p*-anisyl alcohol (**87**), 1-(4-methoxyphenyl)-1,2-propanediol (**88**), and 2-hydroxy (4’-methoxy)-propiophenone (**89**). However, these four compounds were produced in comparatively large amounts, so they certainly contribute to the nematicidal repertoire of the producer (Stadler et al. 1994a). The direct application of nematicidal *Pleurotus* spp. to the soil (Thorn and Barron 1984; Barron and Thorn 1987) should therefore be considered as a potentially cost-effective approach for the biocontrol of phytoparasitic nematodes (Palizi et al. 2009).

Three nematicidal compounds were isolated using bioassay-guided fractionation from a 10-day submerged culture of *Pleurotus eryngii* var. *ferulae* L14, a subspecies associated with *Ferlua communis* subsp. *communis*, the giant fennel (Mang and Figliuolo 2010). Cheimonophyllon E (**90**), a colorless amorphous solid, was obtained from an ethyl acetate extract of the culture filtrate. A yellowish amorphous solid, 5*α*,8*α*-epidioxyergosta-6,22-dien-3-*β*-ol (**91**), and a colorless amorphous solid, 5-hydroxymethyl-furancarbaldehyde (**92**), were detected in the mycelium acetone extract. The LC_50_ values of compounds **90**–**92** against the pine wood nematode (*Bursaphelenchus xylophilus*) were 70.8, 174.6, and 54.7 mg/l, respectively, after 72 h. The LC_50_ values against *P. redivivus* were 125.6, 128.1, and 82.8 mg/l, respectively, after the same exposure (Li et al. 2007).

#### Metabolites from the genera *Coprinus* and *Coprinellus*

The nematophagous fungus *Coprinus comatus* (*Agaricales*, *Coprinaceae*), commonly known as the Shaggy Inkcap or Lawyer’s Wig, forms spiny balls that enhance its nematicidal activity by mechanically damaging the nematode cuticle, ultimately leading to the loss of pseudocoelomic fluid (Luo et al. 2004, 2007). Agar cultures of *C. comatus* C-1 yielded a mixture of nematicidal secondary metabolites after cultivation on potato-dextrose agar at 25 °C for 15 days. Seven compounds were obtained from organic extracts, namely 5-methylfuran-3-carboxylic acid (**93**), 5-hydroxy-3,5-dimethylfuran-2 (5*H*)-one (**94**), 5-hydroxy-3-(hydroxymethyl)-5-methylfuran-2 (5*H*)-one (**95**), 4,6-dihydroxyisobenzofuran-1,3-dione (**96**), 4,6-dihydroxybenzofuran-3 (2*H*)-one (**97**), 4,6-dimethoxyisobenzofuran-1 (3*H*)-one (**98**) and 3-formyl-2,5-dihydroxybenzyl acetate (**99**). Compounds **93** and **94** displayed the most potent nematicidal activity against *Meloidogyne incognita* and *Panagrellus redivivus*, with LD_50_ and LD_90_ values of 100 and 200 μg/ml, respectively, for both compounds (Luo et al. 2007).

Organic extracts of *Coprinus* (now *Coprinellus*) *xanthothrix* (*Agaricales*, *Psathyrellaceae*) 4916 agar cultures yielded three further nematicidal metabolites: xanthonone (**100**), 7,8,11-drimanetriol (**101**) and 2-(1*H*-pyrrol-1-yl)-ethanol (**102**). The LD_50_ values of compounds **100** and **102** were 250 and 125 μg/ml, respectively, against both *M. incognita* and *P. redivivus*, whereas compound **101** was practically inactive (Liu et al. 2008).

#### Metabolites from the genus *Nematoctonus*

*Nematoctonus robustus*, the anamorph of *Hohenbuehelia grisea*^2^ (*Agaricales*, *Pleurotaceae*), is able to trap nematodes conidia, which form sticky knobs upon germination (Dowe 1987). *N. robustus* CBS 945.69 was grown in a fermenter at 24 °C for 11 days until the antimicrobial activities of the extracts did not increase any further. The bioactive principle consisted of dihydropleurotinic acid (**103**) and pleurotin (**104**), two 1,4-naphthoquinone antibiotics, and leucopleurotin (**105**), a precursor thereof. Biosynthesis of pleurotin involves a farnesylhydroquinone intermediate which is further cyclized, rearranged, and oxidized (Gill and Steglich 1987). Compounds **103**–**105** displayed weak antifungal activities and medium-to-weak activities against bacteria and yeasts. None of the three quinones was nematicidal for *C. elegans* (Stadler et al. 1994b); however, effects toward other nematode species have not been reported so far. Notably, pleurotin was shown to act as an inhibitor of the thioredoxin–thioreductase system (Welsh et al. 2003). Subsequently, different species of pleurotin-producing basidiomycetes were investigated, and a fermentation protocol was developed to obtain this anticancer lead metabolite in concentrations >300 mg/l (Shipley et al. 2006). A total synthesis of **104** and **105** was also reported (Hart and Hunag 1988).

**Fig. 1:**
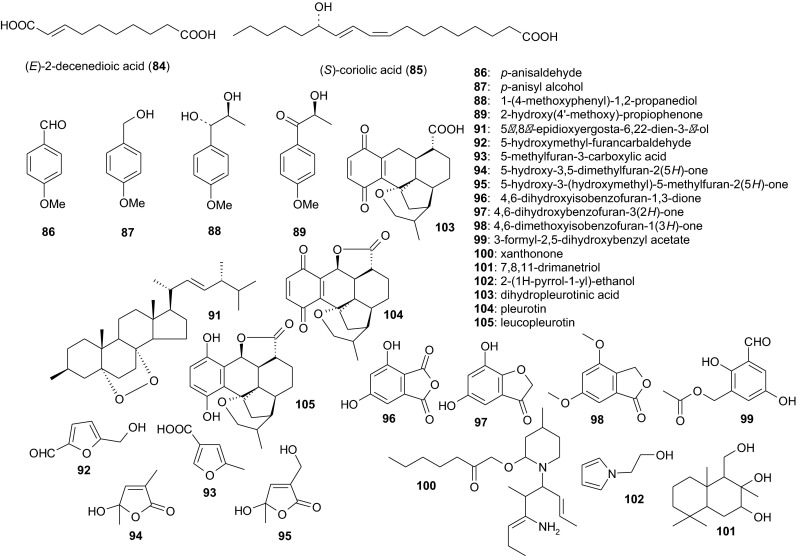
Nematicidal metabolites from nematophagous basidiomycetes. See comments on compounds **103**–**105** within the text

Nematicidal metabolites from nematophagous basidiomycetes as well as compounds **103**–**105** are illustrated in Fig. 1.

## Metabolites from non-nematophagous ascomycetes

### Nematicidal metabolites from *Lachnum papyraceum*

**Fig. 2:**
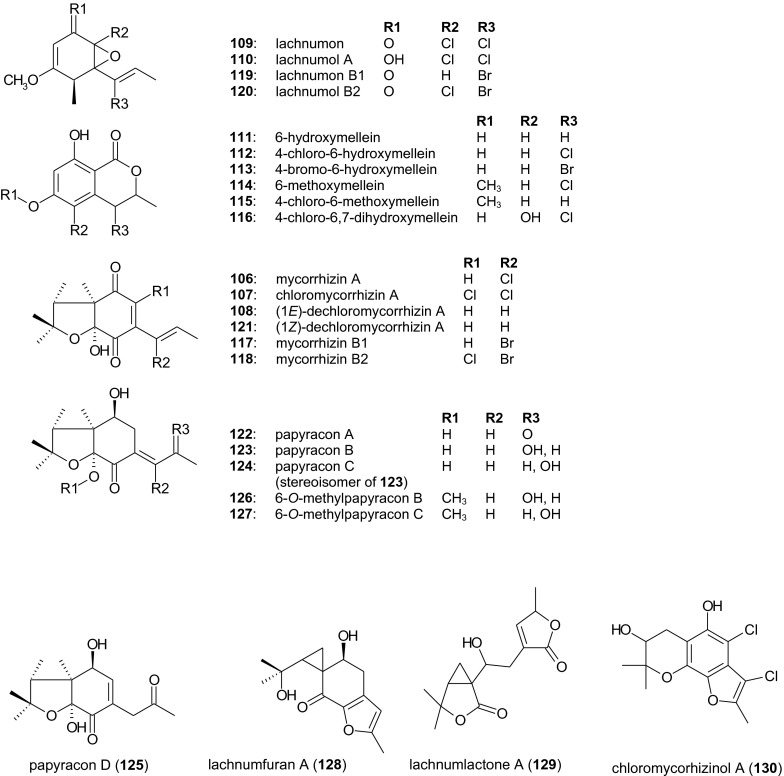
Nematicidal metabolites from the non-nematophagous ascomycete *Lachnum papyraceum*

The wood-inhabiting fungus *L. papyraceum* (*Helotiales*, *Hyaloscyphaceae*) A 48–88 is probably the most thoroughly investigated producer of nematicidal secondary metabolites. Five nematicidal substances were isolated from an 18-day fermentation culture filtrate, all displaying cytotoxic, antimicrobial, and nematicidal activities against *C. elegans* but not *M. incognita* (Stadler et al. 1993a; Anke et al. 1995). Three were identified as the previously known compounds (+)-mycorrhizin A (**106**), (+)-chloromycorrhizin A (**107**) and (1*E*)-dechloromycorrhizin A (**108**), but two novel compounds were isolated as colorless substances, the crystalline lachnumon (**109**) and the oily lachnumol A (**110**), both of which contained a rare chlorinated 5,6-epoxide. Both compounds are therefore highly sensitive to oxygen and acid, and even aqueous or methanolic solutions were highly unstable. Under these conditions, the epoxy group opens to form a reactive cation, leading to further, rapid decomposition (Stadler et al. 1993b). Because chlorine substitution in compounds of terrestrial origin is comparatively rare, the influence of a bromide supplement on the secondary metabolism of strain A 48–88 was investigated. The addition of 5 mM CaCl_2_ and 50 mM CaBr_2_ to the uninoculated fermentation medium led to unexpected changes in the metabolite profile. Notably, chloromycorrhizin A (**107**), (1*E*)-dechloromycorrhizin A (**108**), and lachnumon (**109**) were not detected anymore, and only traces of mycorrhizin (**106**) and lachnumol (**110**) were present. However, six novel metabolites bearing a dihydroisocoumarin (isochroman-1-one) skeleton were identified: 6,8-dihydroxy-3-methylisochroman-1-one (6-hydroxymellein, **111**), 4-chloro-6-hydroxymellein (**112**), 4-bromo-6-hydroxymellein (**113**), 6-methoxymellein (**114**), 4-chloro-6-methoxymellein (**115**), and 4-chloro-6,7-dihyroxymellein (**116**). All six compounds were only weakly nematicidal (Stadler et al. 1995a, b). The addition of CaBr_2_ following the detection of (1*E*)-dechloromycorrhizin A (**108**) after 10 days of fermentation resulted in further diversification of the secondary metabolite profile. Brominated analogs named mycorrhizin B1 (**117**), mycorrhizin B2 (**118**), lachnumon B1 (**119**), and lachnumon B2 (**120**) were identified, and their activity was found to be slightly lower than their chlorinated counterparts (Stadler et al. 1995c, d). In addition to a stereoisomer of compound **108**, four non-halogenated compounds were isolated, namely (1*Z*)-dechloromycorrhizin A (**121**) and the three novel mycorrhizin-related analogs papyracons A, B, and C (**122**–**124**) which showed mutagenic activity in the Ames test (Stadler et al. 1995c, e). Further minor compounds were isolated with weak nematicidal activity against *C. elegans* (Shan et al. 1996): papyracon D (**125**), 6-*O*-methylpapyracon B (**126**), 6-*O*-methylpapyracon C (**127**), lachnumfuran A (**128**), lachnumlactone A (**129**), and chloromycorrhizinol A (**130**). This detailed analysis of the “nematicidal fraction” of *L. papyraceum* A 48–88 also revealed the susceptibility of *C. elegans* to a broad range of metabolites. Bioassay-guided screening for nematicidal compounds should therefore be carried out using economically important phytoparasitic nematodes (Anke et al. 1995). The impressive arsenal of nematicidal metabolites from the non-nematophagous ascomycete *L. papyraceum* is summarized in Fig. 2.

### Bulgarialactones from *Bulgaria inquinans* A 40–94

The black bulgar (*Bulgaria inquinans*), a saprotrophic ascomycete (*Phacidiales*, *Phacidiaceae*), grows on the bark of decaying deciduous trees and logs, preferably on oak. An organic extract of fruiting bodies yielded three azaphilones, named bulgarialactones A, B, and C (**131**–**133**), but only compound **132** could be isolated in sufficient quantities for further analysis. The mycelia of an 11-day submerged culture of strain A 40–94 yielded compounds **131**–**133** as dark red oils, whereas organic extracts of the culture filtrate yielded only compound **132** in preparative amounts. The LD_50_ values of compounds **131** and **132** against *C. elegans* were 5 and 10–25 μg/ml, respectively, whereas compound (**133**) could not be tested due to its instability and low yield (Stadler et al. 1995f).

### Thermolides from *Talaromyces thermophilus*^3^ YM 3–4

Submerged cultures of the thermophilc fungus *Talaromyces thermophilus* YM 3–4 were grown for 21 days at 45 °C, yielding six colorless oils, named thermolides A–F (**134**–**139**). These provided the first evidence for a hybrid polyketide synthase non-ribosomal peptide synthetase (PKS-NRPS) of fungal origin (Niu et al. 2014).^4^ All thermolides feature an unusual 13-membered lactam-bearing macrolactone ring system. Thermolides A (**134**) and B (**135**) exhibited strong nematicidal activities with LC_50_ values against *M. incognita*, *Bursaphelenchus xylophilus*^5^ and *P. redivivus* as high as those of the avermectin standard, ranging from 0.5 to 1.0 μg/ml. Thus, thermolides A and B but also the less bioactive thermolides C (**136**) and D (**137**)^6^ may serve as lead candidates for the development of new biological nematicides (Guo et al. 2012).

### Paraherquamides from *Penicillium charlesii* ATCC 20841

Seven oxindole alkaloids, paraherquamides A–G (**141**–**147**), were isolated from 7- or 14-day static cultures of *Penicillium charlesii* ATCC 20841 grown at 25 °C. The major compound paraherquamide A (**141**) was also the most active one, with an LD_50_ value of 2.5 μg/ml against *C. elegans* (**141**). The LD_50_ values of the other compounds ranged from 6 μg/ml (**145**) to 160 μg/ml (**144**). Broad-spectrum activity was observed against the three pathogenic nematodes *Haemonchus contortus*, *Trichostrongylus colubriformis*, and *T. sigmodontis*, each of them located in a distinct part of the gastrointestinal tract of the gerbil, *Meriones unguiculatus* (Ostlind et al. 2006). The insecticidal activity of paraherquamides against the milkweed bug, *Oncopeltus fasciatus* (*Hemiptera*, *Lygaeidae*), has also been reported (López-Gresa et al. 2006).

### Cochlioquinone A from *Bipolaris sorokiniana*

*B. sorokiniana* (syn. *Cochliobolus sativus*, *Pleosporaceae*, *Pleosporales*) is one of the most notorious plant pathogens in warmer climates, and as the cause of southern leaf blotch, seedling blight, crown rot, node infections, head blight and black point on kernels; it is regarded as the economically most important foliar pathogen of wheat (Manamgoda et al. 2014). Static cultures in vermiculite-containing medium were incubated for 14 days at 25 °C producing the yellow, crystalline *p*-benzoquinone derivative cochlioquinone A (**148**). This caused the immobilization of 50 % of a *C. elegans* population after 16 h at a concentration of 135 μM (Schaeffer et al. 1990). Cochlioquinone A was also obtained from *B. leersiae* (Barrow and Murphy 1972), which is a pathogen of *Leersia* and *Setaria* spp. (*Poaceae*, Manamgoda et al. 2014).

### Nematicidal ophiobolins

Approximately 30 C_25_ sesterterpenoids bearing a tricyclic 5-8-5 ring system (ophiobolins) have been isolated from fungi. Most of the producers are members of the genus *Bipolaris* (*Pleosporales*, *Pleosporaceae*), which include economically important phytopathogens such as *B. oryzae* (syn. *Cochliobolus miyabeanus*), the brown spot pathogen of rice *B. maydis* (*C. heterostrophus*) that causes southern corn leaf blight, and *B. sorghicola*, which causes leaf spot in sorghum. Even so, ophiobolin K (**149**) was initially isolated from *Aspergillus ustus* JP 118 growing in a roller jar on a solid vermiculite-containing medium for 28 days at 25 °C. This caused the immobilization of 50 % of a *C. elegans* population after 16 h at a concentration of 10 μg/ml, whereas 6-epiophiobolin K (**150**) was inactive (Singh et al. 1991). Ophiobolins C (**151**) and M (**152**) were isolated from the necrotrophic pathogen *B. maydis* grown in static culture for 14 days at 25 °C. The LD_50_ values of compounds **149**, **151**, and **152** against *C. elegans* were 26, 5, and 13 μM, respectively. Ophobolins were shown to non-competitively inhibit the binding of ivermectin to membrane preparations from *C. elegans*, which accounts for an interaction at the ivermectin binding site (Tsipouras et al. 1996).^7^ The practical application of ophiobolins may be limited by their instability (Yun et al. 1988) and other diverse bioactivities (Au et al. 2000). For example, some ophiobolins are strongly phytotoxic, whereas others were harmless to plants (Yun et al. 1988; Evidente et al. 2006a, b). No structure-activity data are yet available to evaluate the relationship between the nematicidal and phytotoxic activities of these compounds.

### Bursaphelocides from an anamorphic fungus

A taxonomically unidentified, sterile fungus (strain D1084) isolated from plant debris and grown in submerged culture for 6 days at 27 °C was shown to produce the cyclodepsipeptides bursaphelocides A and B (**155**, **156**). Both compounds contain 2-hydroxy-3-methylpentanoic acid, isoleucine, *N-*methylvaline, *N*-methylalanine and *β*-alanine, but they differ in that compound **155** also contains proline, whereas in compound **156**, this residue is 4-methylproline.^8^ The “cotton ball on fungal mat method” was used for bioassay-guided fractionation of the culture broth. Compounds **155** and **156** caused >96 and >98 % mortality, respectively, when added to cultures of *B. xylophilus* at a concentration of 100 μg/ml per ball. Insecticidal activity was observed against *Drosophila melanogaster* larvae as well as weak phytotoxic activity in an alfalfa (*Medicago sativa*) seed germination test (Kawazu et al. 1993).

### A δ-lactone from *Trichoderma* sp. YMF 1.00416

The simple δ-lactone 6-*n*-pentyl-2*H*-pyran-2-one (6-PAP) (**157**) represents the characteristic odoriferous volatile (“coconut flavor”) of several *Trichoderma* spp. (*Hypocreales*, *Hypocreaceae*). A list of 77 isolates from 8 phylogenetically verified PAP-producing species has recently been compiled (Jeleń et al. 2014). Compound **157** is best known for its antagonistic activity toward a number of economically important phytopathogenic fungi (Gräfenhan 2006; Reino et al. 2008). More recently, organic extracts of the soil-borne fungus *Trichoderma* sp. YMF 1.00416 from a submerged culture grown at 28 °C were also tested for nematicidal activity. After 48 h exposure, the LD_50_ values against *P. redivivus*, *C. elegans*, and *B. xylophilus* were 69, 71, and 94 μg/ml, respectively (Yang et al. 2012). Other *Trichoderma*-derived pyrones such as 6-(1’-pentenyl)-2*H*-pyran-2-one, massoialactone, δ-decalactone, and viridepyronone should therefore be screened for nematicidal activity too (Reino et al. 2008).

### Endophytic ascomycetes producing 3-hydroxypropionic acid

**Fig. 3:**
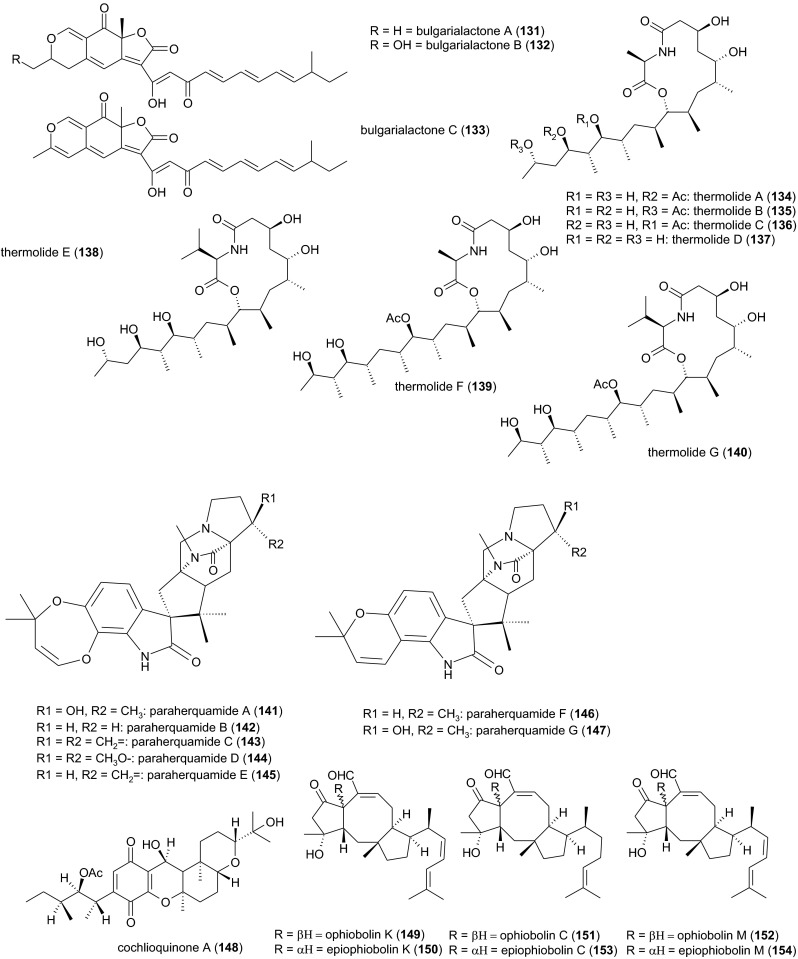

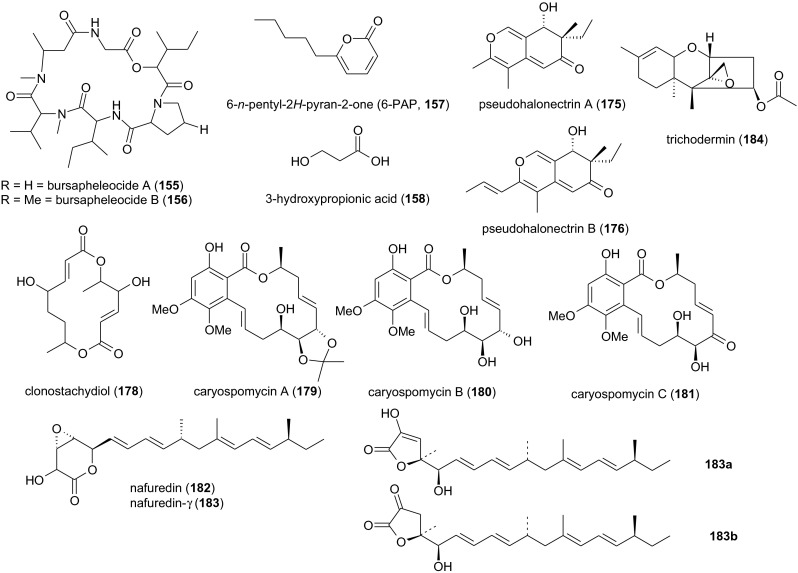
Nematicidal metabolites from other non-nematophagous ascomycetes

Submerged cultures of a number of endophytic fungi were screened for potential nematicidal activity against *B. xylophilus* using bioassay-guided fractionation. Five strains with the highest activities were used for the isolation and structural elucidation of the bioactive principles, including *Phomopsis phaseoli* (*Diaporthaceae*, *Diaporthales*) and *Melanconium betulinum* (*Melanconidaceae*, *Diaporthales*). However, the only nematicidal metabolite in all five isolates was identified as 3-hydroxypropionic acid (**158**). Notably, both of the species listed above may live either as plant pathogens or harmless endophytes (Schwarz et al. 2004). Because phytotoxic fungal isolates must not be used for integrated pest management, the pure compound should instead be considered for biocontrol applications. The structures of nematicidal metabolites from non-nematophagous ascomycetes are summarized in Fig. 3.

### Metabolites from non-nematophagous basidiomycetes

#### Metabolites from *Cheimonophyllum candidissimum* TA 8644

Six bisabolane-type sesquiterpenoids were isolated from a culture filtrate of the xylophagous fungus *Cheimonophyllum candidissimum* (*Agaricales*, *Cyphellaceae*) after 168 h of submerged fermentation, namely cheimonophyllon E (**90**), cheimonophyllons A–D (**159**–**162**) and cheimonophyllal (**163**). The LD_50_ values of the compounds against *C. elegans* were 10 μg/ml (compounds **159** and **162**), 25 μg/ml (compounds **160** and **163**), 50 μg/ml (compound **161**)^9^ and >100 μg/ml (compound **93**). Compound **159** was weakly mutagenic in the Ames test but no comparable data are available for the others. No phytotoxicity was observed, but the stability of compounds **159**, **160**, **162**, and **163** is limited by their reactivity (Stadler et al. 1994c, d). Asymmetric total synthesis of (+)-cheimonophyllon E (**90**) and (+)-cheimonophyllal (**163**) has been reported (Takao et al. 2002). An additional minor compound, the nematicidal *p*-menthan-type monoterpene 1,2-dihydroxymintlactone (**164**), was subsequently obtained as a colorless oil from *C. candidissimum* TA 8644. Its LD_50_ value against *C. elegans* was 25 μg/ml (Stadler et al. 1995g).

#### Omphalotins from *Omphalotus olearius* TA 90170

Mycelia from submerged cultures of the jack-o’-lantern mushroom *Omphalotus olearius* (*Agaricales*, *Omphalotaceae*) yielded nine nematicidal cyclic dodecapeptides that were not present in the fruiting bodies. The main compound, omphalotin A (**165**), is a colorless oil that remains stable during isolation and storage. Remarkably, its LD_50_ against the plant-parasitic *M. incognita* was 2 μg/ml, which is ten times more potent than the ivermectin standard. The saprotrophic nematode *C. elegans* was 35-fold less susceptible. Compound **165** was shown to protect cucumber and lettuce cultures from nematodes, with no evidence of additional phytotoxic, insecticidal, or antimicrobial activities. Cytotoxic effects were comparatively weak (Sterner et al. 1997; Mayer et al. 1997, 1999). Compound **165** contains a high proportion of methylated l-amino acids including sarcosine (methylglycine), methylvaline, and methylisoleucine (Sterner et al. 1997; Büchel et al. 1998). Three minor compounds, omphalotins B, C, and D (**166**–**168**), were obtained after prolonged fermentation. Their nematicidal activity was reported to be similar to omphalotin A but no data were presented (Büchel et al. 1998). Monokaryotic strains, which have been obtained from *O. olearius* TA 90170 protoplasts, yielded five additional hydroxylated compounds, omphalotins E–I (**169**–**173**), after 9 days of fermentation. Their nematicidal activity against *M. incognita* was highly selective, with LD_50_ values between 0.5 and 2.0 μg/ml (Liermann et al. 2009). One future challenge is to optimize fermentation conditions to improve the low yields of these compounds. In the meantime, a high-yielding method for solid-phase synthesis has been developed for compound **165** and other *N*-alkylated peptides using racemization-free triphosgene-mediated couplings (Thern et al. 2002).

#### Illinitone A from *Limacella illinita* strain 99049

**Fig. 4:**
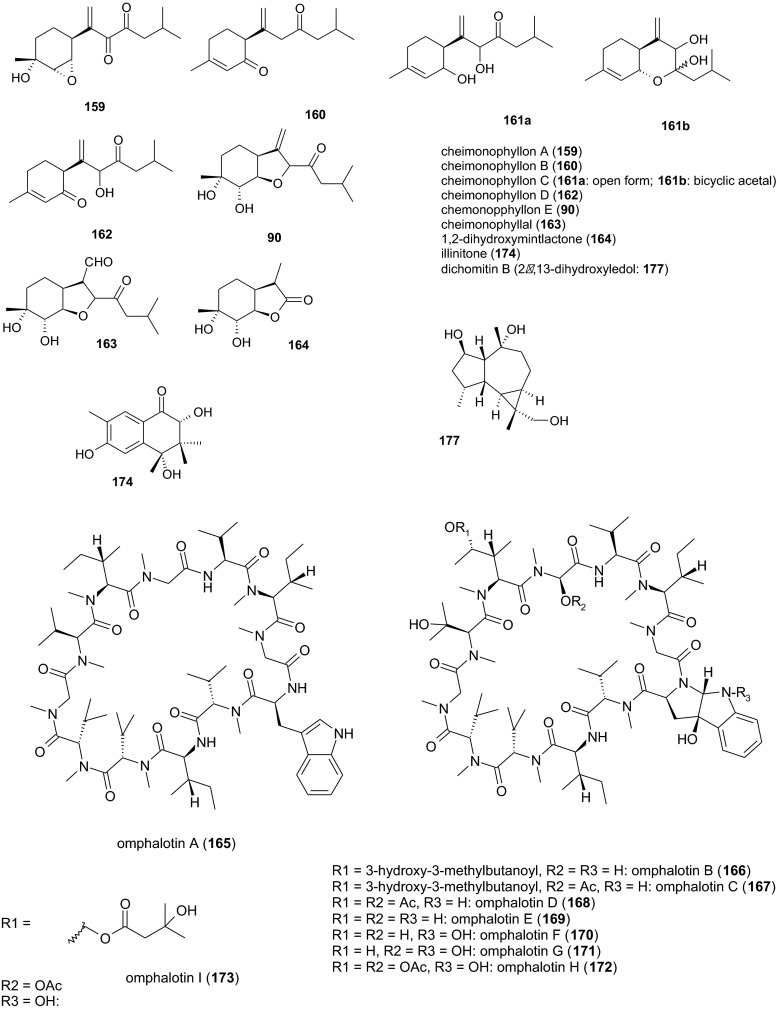
Nematicidal metabolites from non-nematophagous basidiomycetes

Submerged cultures of the Dripping Slimecap *Limacella illinita* (*Agaricales*, *Amanitaceae*) yielded, after approximately 21 days, the colorless oil illinitone A (**174**). The LD_50_ value of this terpenoid compound against *C. elegans* was 25 μg/ml. High concentrations (333 μg/ml) inhibited shoot and root growth in the garden cress (*Lepidium sativum*) and foxtail millet (*Setaria italica*) by 60 % (Gruhn et al. 2007). Nematicidal metabolites from non-nematophagous basidiomycetes are summarized in Fig. 4.

#### Outlook and perspectives

More than 30,000 natural products have been isolated from fungi (Bérdy 2012), but fewer than 300 nematicidal compounds have been confirmed, representing just 280 producing species distributed over 150 genera (Laatsch 2014; Li and Zheng 2014). The screening of culture collections for nematicide-producing fungi could therefore yield more useful compounds than libraries of previously isolated natural products. The chemical structures of nematicidal metabolites are highly diverse, ranging from simple fatty acids and other organic acids to pyrones, lactones, benzoquinones, anthraquinones, furans, alkaloids, cyclodepsipeptides, peptaibiotics, and hybrid structures such as lactam-bearing macrolactones. It is therefore impossible to predict whether either a given fungal species or a particular fungal metabolite is likely to be nematicidal, and the activity against different nematode species may also vary. It is therefore essential to screen fungi and their metabolites against multiple economically important nematode species (Table 1), including common phytoparasites and nematodes that parasitize animals (e.g., *H. contortus*). The established model species *C. elegans* is often exquisitely sensitive toward nematicides, even primary metabolites such as fatty acids (Stadler et al. 1994a; Anke et al. 1995), although exceptions include oligosporon (**2**), which is inactive against *C. elegans* but moderately active against *H. contortus* (Anderson et al. 1995).

**Table 1:** Further lead metabolites from fungi exhibiting pronounced activity against plant-parasitic and intestinal nematodes

Substance	Class	Producer	Effects	References
Pseudohalonectrin A (**175**), B (**176**)	Azaphilones	*Pseudohalonectria adversaria* YMF1.01019 (*Magnaporthaceae*, *Magnaporthales*)	>50 % mortality against *B. xylophilus* after 24 h (100 μg/ml)	Dong et al. 2006
2*β*,13-dihydroxyledol (= dichomitin B, **177**)	Sesquiterpene	*Dichomitus squalens* (*Polyporales*, *Polyporaceae*)	LC_50_ = 35,6 μg/ml against *B. xylophilus* after 24 h	Huang et al. 2004
Clonostachydiol (**178**)	Macrodiolide	*Clonostachys cylindrospora* FH-A6607 (*Hypocreales*, *Bionectriaceae*)	80–90% reduction of fecal *H. contortus* in lambs after 14 days, following subcutaneous admission of 2.5 mg/kg clonostachydiol	Grabley et al. 1993
Caryopsomycins A–C (**179**–**181**)	Resorcylic acid lactones	*Caryospora callicarpa* YMF1.01026 (*Pleosporales*, *Zopfiaceae*)	LC_50_ against *B. xylophilus* [μg/ml] after 36 h: 103.1 (**179**), 105.8 (**180**), and 105.1 (**181**)	Dong et al. 2007
Nafuredin (**182**)	Epoxy-*δ*-lactone with olefinic side chain	*Aspergillus niger* FT-0554 (*Eutotiales*, *Aspergillaceae*)	>90% reduction of *H. contortus* eggs after 11 days, following one single treatment of sheep with 2 mg/kg nafuredin p.o.; complete suppression of egg development following a second treatment 1 week after first administration	Ui et al. 2001; Ōmura et al. 2001
Nafuredin-*γ* (**183**)	*γ*-lactone with olefinic side chain	*Aspergillus niger* FT-0554	92 % reduction of *H. contortus* eggs 11 days after the second treatment, conducted 3 weeks after first administration	Shiomi et al. 2005

In the second part of this review, 101 substances from nematophagous basidiomycetes and non-nematophagous fungi were introduced, some of which exhibit pronounced nematicidal activity.^10^ Thermolides A (**134**) and B (**135**) displayed potent nematicidal activity against *M. incognita*, *B. xylophilus*, and *P. redivivus*, comparable to that of the avermectin standard, but it remains difficult to produce large amounts of these compounds because the producers are thermophilic and cannot grow efficiently at temperatures below 45°C, so cultivation conditions will need to be optimized. Other potent fungal nematicides discussed herein have only been isolated in minute quantities. This may reflect suboptimal fermentation conditions, as observed for the omphalotins (**165**–**173**), or physicochemical instability, as observed for epoxidized lachnumon (**109**, **119**, **120**) and lachnumol derivatives (**110**), bulgarialactones (**131**–**133**), and ophiobolins K (**149**), M (**151**), and C (**152**).

Another challenge that must be addressed is that some nematicide-producing fungi are obligate phytopathogens (e.g., *Bipolaris* spp.), whereas others are facultative phytopathogens that may also exist as endophytes. In these cases, the producers cannot be used as biocontrol agents, and the nematicidal compounds they biosynthesize must be isolated, e.g., cochlioquinone A (**148**) and 3-hydroxypropionic acid (**158**). Yang et al. (2010) have even suggested that the nematicidal mycotoxin trichodermin (**184**) could be isolated from *Trichoderma* strains producing it, but the use of mycotoxigenic fungi or pure mycotoxins in biocontrol had been discussed and argued against by Degenkolb et al. (2008) and Chaverri et al. (2015). Mycorrhizin A (**106**) and some of its derivatives from *L. papyraceum* (**107**, **108**, **117**, **118**, **121**) as well as cheimonophyllon A (*159*) showed at least weak mutagenic activity in the Ames test.

Several promising examples of secondary metabolites from non-nematophagous fungi have also been discussed. Glasshouse and field trials with phylogenetically verified *Trichoderma* species producing either 6-PAP (**157**) (Gräfenhan 2006; Jeleń et al. 2014) or structurally related simple pyrones (Reino et al. 2008) should be conducted because their combined nematicidal and fungicidal properties are highly desirable for agricultural applications. Thermolides A (**134**) and B (**135**), omphalotins (**165**–**173**), ophiobolins^11^ (**149**, **151**, **152**), bursaphelocides A (**155**) and B (**156**), illinitone A (**174**), pseudohalonectrins A (**175**) and B (**176**), dichomitin B (**177**), and caryopsomycins A–C (**179**–**181**) are excellent candidates or lead compounds for the development of biocontrol strategies for phytopathogenic nematodes, whereas paraherquamides (**141**–**147**), clonostachydiol (**178**), and nafuredins (**182**/**183**) offer promising leads for the development of formulations against the intestinal nematodes of ruminants (Table 1).

## Footnotes

1 *Incertae sedis*, formerly belonging to the traditional *Zygomycota*.

2 *Hohenbuehelia* spp. capture nematodes by hourglass-shaped sticky traps (Dowe 1987). The anamorph-teleomorph combination *Nematoctonus*/*Hohenbuehelia* forms a monophyletic, yet diverse clade within the *Pleurotaceae* (Koziak et al. 2007).

3 The fungus has recently been reclassified as *Thermomyces dupontii. Penicillium dupontii* and *Talaromyces thermophilus* are used synonymously. Both thermolide-producing species mentioned here belong to the *Trichocomaceae*, according to their ITS sequences (Houbraken et al. 2014).

4 A related compound, thermolide G (**140**), has recently been isolated from *Thermomyces lanuginosus* (strains G5 and ATCC 200065), but no information about its nematicidal activity is available (Niu et al. 2014).

5 Mis-spelled as *Bursaphelench**e**s**siylopilus* by Guo et al. (2012).

6 No information concerning the nematicidal activities of thermolides E and F is available because only minute amounts have been isolated (Guo et al. 2012).

7 Notably, 6-epiophiobolin C (**153**) and 6-epiophiobolin M (**154**) did not display any nematicidal activity (Tsipouras et al. 1996).

8 The configuration of the amino acids has not yet been determined.

9 In solutions of cheimonophyllon C (**161**), a 1:1 mixture of the open form (**161a**) and the bicyclic acetal (**161b**) was present (Stadler et al. 1994c).

10 Other nematicidal metabolites reported in the literature are not considered here because concentrations of 200–1000 μg/ml are required to achieve nematicidal effects.

11 The nematicidal activity of most of the natural ophiobolins remains to be studied.

## References

[CR1] Anderson MG, Jarmin TB, Rickards RW (1995). Structures and absolute configurations of antibiotics of the oligosporon group from the nematode-trapping fungus *Arthrobotrys oligospora*. J Antibiot.

[CR2] Anke H, Stadler M, Mayer A, Sterner O (1995). Secondary metabolites with nematicidal and antimicrobial activity from nematophagous fungi and *Ascomycetes*. Can J Bot.

[CR3] Au TK, Chick WSH, Leung PC (2000). The biology of the ophiobolins. Life Sci.

[CR4] Barron GL (1977). The nematode-destroying fungi. Topics in mycobiology No. 1.

[CR5] Barron GL, Thorn RG (1987). Destruction of nematodes by species of *Pleurotus*. Can J Bot.

[CR6] Barrow KD, Murphy WS (1972). The structures of alboleersin and luteoleersin; the identity of luteoleersin with cochlioquinone A. J Chem Soc Perkin Trans.

[CR7] Bérdy J (2012). Thoughts and facts about antibiotics: where we are now and where we are heading. J Antibiot.

[CR8] Büchel E, Martini U, Mayer A, Anke H, Sterner O (1998). Omphalotins B, C and D, nematicidal cyclopeptides from *Omphalotus olearius* absolute configuration of omphalotin A. Tetrahedron.

[CR9] Chaverri P, Branco-Rocha F, Jaklitsch W, Gazis R, Degenkolb T, Samuels GJ (2015). Systematics of the *Trichoderma harzianum* species complex and the re-identification of commercial biocontrol strains. Mycologia.

[CR10] Degenkolb T, Dieckmann R, Nielsen KF, Gräfenhan T, Theis C, Zafari D, Chaverri P, Ismaiel A, Brückner H, von Döhren H, Thrane U, Petrini O, Samuels GJ (2008). The *Trichoderma brevicompactum* clade: a separate lineage with new species, new peptaibiotics, and mycotoxins. Mycol Prog.

[CR11] Dong J, Zhou J, Li R, Zhou W, Zhu Y, Huang R, Zhang K (2006). New nematicidal azaphilones from the aquatic fungus *Pseudohalonectria adversaria* YMF1.01019. FEMS Microbiol Lett.

[CR12] Dong J, Zhu Y, Song H, Li R, He H, Liu H, Huang R, Zhou Y, Wang L, Cao Y, Zhang K (2007). Nematicidal resorcylides from the aquatic fungus *Caryospora callicarpa* YMF1.01026. J Chem Ecol.

[CR13] Evidente A, Andolfi A, Cimmino A, Vurro M, Fracchiolla M, Charudattan R (2006). Herbicidal potential of ophiobolins produced by *Drechslera gigantea*. J Agric Food Chem.

[CR14] Evidente A, Andolfi A, Cimmino A, Vurro M, Fracchiolla M, Charudattan R, Motta A (2006). Ophiobolin E and 8-epi-ophiobolin J produced by *Drechslera gigantea*, a potential mycoherbicide of weedy grasses. Phytochemistry.

[CR15] Gill M, Steglich W (1987). Pigments of fungi (macromycetes). Fortschr Chem Org Naturst.

[CR16] Grabley S, Hammann P, Thiericke R, Wink J, Philips S, Zeeck A (1993). Secondary metabolites by chemical screening. 21. Clonostachydiol, a novel anthelmintic macrodiolide from the fungus *Clonostachys cylindrospora* (strain FH-A 6607). J Antibiot.

[CR17] Gräfenhan T (2006) Epidemiology and biological control of latent grapevine trunk diseases. Dissertation, Humboldt University of Berlin

[CR18] Gruhn N, Schoettler S, Sterner O, Anke T (2007). Biologically active metabolites from the basidiomycete *Limacella illinita* (Fr.) Murr. Z Naturforsch.

[CR19] Guo J-P, Zhu C-Y, Zhang C-P, Chu Y-S, Wang Y-L, Zhang J-X, Wu D-K, Zhang K-Q, Niu X-M (2012). Thermolides, potent nematocidal PKS-NRPS hybrid metabolites from thermophilic fungus *Talaromyces thermophilus*. J Am Chem Soc.

[CR20] Hart DJ, Hunag H-C (1988). Total synthesis of (±)-pleurotin and (±)-dihydropleurotin acid. J Am Chem Soc.

[CR21] Houbraken J, de Vries RP, Samson RA (2014). Modern taxonomy of biotechnologically important *Aspergillus* and *Penicillium* species. Adv Appl Microbiol.

[CR22] Huang Z, Dan Y, Huang Y, Lin L, Li T, Ye W, Wie X (2004). Sesquiterpenes from the mycelial cultures of *Dichomitus squalens*. J Nat Prod.

[CR23] Jeleń H, Błaszczyk L, Chełkowski J, Rogowicz K, Strakowska J (2014). Formation of 6-n-pentyl-2*H*-pyran-2-one (6-PAP) and other volatiles by different *Trichoderma* species. Mycol Prog.

[CR24] Kawazu K, Murakami T, Ono Y, Kanzaki H, Kobayashi A, Mikawa T, Yoshikawa N (1993). Isolation and characterization of two novel nematicidal depsipeptides from an imperfect fungus, strain DI084. Biosci Biotechnol Biochem.

[CR25] Kirk PM, Cannon PF, Minter DW, Stalpers JA (2008). Dictionary of the fungi.

[CR26] Koziak AT, Cheng KC, Thorn RG (2007). Phylogenetic analyses of *Nematoctonus* and *Hohenbuehelia* (*Pleurotaceae*). Can J Bot.

[CR27] Kwok OCH, Plattner R, Weisleder D, Wicklow DT (1992). A nematicidal toxin from *Pleurotus ostreatus* NRRL 3526. J Chem Ecol.

[CR28] Laatsch H (2014). Antibase 2014 SciDex v. 1.2.495—the natural compounds identifier.

[CR29] Li G-H, Zhang K-Q, Hyde KD, Zhang K-Q (2014). Nematode-toxic fungi and their nematicidal metabolites. Nematode-trapping fungi.

[CR30] Li G, Wang X, Zheng L, Li L, Huang R, Zhang K (2007). Nematicidal metabolites from the fungus *Pleurotus ferulae* Lenzi. Ann Microbiol.

[CR31] Liermann JC, Opatz T, Kolshorn H, Antelo L, Hof C, Anke H (2009) Omphalotins E–I, five oxidatively modified nematicidal cyclopeptides from *Omphalotus olearius*. Eur J Org Chem 1256–1262

[CR32] Liu YJ, Liu Y, Zhang KQ (2008). Xanthothone, a new nematicidal N-compound from *Coprinus xanthothrix*. Chem Nat Compd.

[CR33] López-Gresa MP, González MC, Ciavatta L, Ayala I, Moya P, Primo J (2006). Insecticidal activity of paraherquamides, including paraherquamide H and paraherquamide I, two new alkaloids isolated from *Penicillium cluniae*. J Agric Food Chem.

[CR34] Luo H, Mo M, Huang X, Li X, Zhang K (2004). *Coprinus comatus*: a basidiomycete fungus forms novel spiny structures and infects nematode. Mycologia.

[CR35] Luo H, Liu Y, Fang L, Li X, Tang N, Zhang K (2007). *Coprinus comatus* damages nematode cuticles mechanically with spiny balls and produces potent toxins to immobilize nematodes. Appl Environ Microbiol.

[CR36] Manamgoda DS, Rossman AY, Castlebury LA, Crous PW, Madrid H, Chukeatirote E, Hyde KD (2014). The genus *Bipolaris*. Stud Mycol.

[CR37] Mang SM, Figliuolo G (2010). Species delimitation in *Pleurotus eryngii* species-complex inferred from ITS and EF-1*α* gene sequences. Mycology.

[CR38] Mayer A, Anke H, Sterner O (1997). Omphalotin, a new cyclic peptide with potent nematicidal activity from *Omphalotus olearius*. I. fermentation and biological activity. Nat Prod Lett.

[CR39] Mayer A, Kilian M, Hoster B, Sterner O, Anke H (1999). *In*-*vitro* and *in*-*vivo* nematicidal activities of the cyclic dodecapeptide omphalotin A. Pestic Sci.

[CR40] Morris EE, Hajek AE (2014). Eat or be eaten: fungus and nematode switch off as predator and prey. Fungal Ecol.

[CR41] Niu X, Chen L, Yue Q, Wang B, Zhang J, Zhu C, Zhang K, Bills GF, An Z (2014). Characterization of thermolide biosynthetic genes and a new thermolide from sister thermophilic fungi. Org Lett.

[CR42] Ōmura S, Miyadera H, Ui H, Shiomi K, Yamaguchi Y, Masuma R, Nagamitsu T, Takano D, Sunazuka T, Harder A, Kölbl H, Namikoshi M, Miyoshi H, Sakamoto K, Kita K (2001). An anthelmintic compound, nafuredin, shows selective inhibition of complex I in helminth mitochondria. Proc Acad Natl Sci USA.

[CR43] Ostlind DA, Cifelli S, Mickle WG, Smith SK, Ewanciw DV, Rafalko B, Felcetto T, Misura A (2006) Evaluation of broad-spectrum anthelmintic activity in a novel assay against *Haemonchus contortus**Trichostrongylus colubriformis *and *T. sigmodontis* in the gerbil *Meriones unguiculatus*. J Helminthol 80:393–396 10.1017/joh2006371 17125549

[CR44] Palizi P, Goltapeh EM, Pourjam E, Safaie N (2009). Potential of oyster mushrooms for the biocontrol of sugar beet nematode (*Heterodera schachtii*). J Plant Protect Res.

[CR45] Reino JL, Guerrero RF, Hernández-Galán R, Collado IG (2008). Secondary metabolites from species of the biocontrol agent *Trichoderma*. Phytochem Rev.

[CR46] Schaeffer JM, Frazier EG, Bergstrom AR, Williamson JM, Liesch JM, Goetz MA (1990). Cochlioquinone A, a nematocidal agent which competes for specific [^3^H]ivermectin binding sites. J Antibiot.

[CR47] Schwarz M, Köpcke B, Weber RWS, Sterner O, Anke H (2004). 3-Hydroxypropionic acid as a nematicidal principle in endophytic fungi. Phytochemistry.

[CR48] Shan R, Stadler M, Sterner O, Anke H (1996). New metabolites with nematicidal and antimicrobial activities from the ascomycete *Lachnum papyraceum* (Karst.) Karst. VIII. Isolation, structure determination and biological activities of minor metabolites structurally related to mycorrhizin A. J Antibiot.

[CR49] Shiomi K, Ui H, Suzuki H, Hatano H, Nagamitsu T, Takano D, Miyadera H, Yamashita T, Kita K, Miyoshi H, Harder A, Tomoda H, Ōmura S (2005). A *γ*-lactone form nafuredin, nafuredin-*γ*, also inhibits helminth complex I. J Antibiot.

[CR50] Shipley SM, Barr AL, Graf SJ, Collins RP, McCloud TG, Newman DJ (2006). Development of a process for the production of the anticancer lead compound pleurotin by fermentation of *Hohenbuehelia atrocaerulea*. J Ind Microbiol Biotechnol.

[CR51] Singh SB, Smith JL, Sabnis GS, Dombrowski AW, Schaeffer JM, Goetz MA, Bills GF (1991). Structure and conformation of ophiobolin K and 6-epiophiobolin K from *Aspergillus ustus* as a nematocidal agent. Tetrahedron.

[CR52] Stadler M, Anke H, Arendholz WR, Hansske F, Anders U, Sterner O, Bergquist K-E (1993). Lachnumon and lachnumol a, new metabolites with nematicidal and antimicrobial activities from the ascomycete *Lachnum papyraceum* (Karst.) karst. I. Producing organism, fermentation, isolation and biological activities. J Antibiot.

[CR53] Stadler M, Anke H, Bergquist KE, Sterner O (1993b) Lachnumon and lachnumol a, new metabolites with nematicidal and antimicrobial activities from the ascomycete *Lachnum papyraceum* (Karst.) Karst. II. Structural elucidation. J Antibiot 46:968–971 10.7164/antibiotics.46.968 8344879

[CR54] Stadler M, Mayer A, Anke H, Sterner O (1994). Fatty acids and other compounds with nematicidal activity from cultures of basidiomycetes. Planta Med.

[CR55] Stadler M, Sheldrick WS, Dasenbrock J, Steglich W, Anke H (1994). Antibiotics from the nematode-trapping basidiomycete *Nematoctonus robustus*. Nat Prod Lett.

[CR56] Stadler M, Anke H, Sterner O (1994). New nematicidal and antimicrobial compounds from the basidiomycete *Cheimonophyllum candidissimum* (Berk & Curt.) Sing. I. Producing organism, fermentation, isolation, and biological activities. J Antibiot.

[CR57] Stadler M, Anke H, Sterner O (1994). Six new antimicrobial and nematicidal bisabolanes from the basidiomycetes *Cheimonophyllum candidissimum*. Tetrahedron.

[CR58] Stadler M, Anke H, Sterner O (1995a) Metabolites with nematicidal and antimicrobial activities from the ascomycete *Lachnum papyraceum* (Karst.) Karst. III. Production of novel isocoumarin derivatives, isolation, and biological activities. J Antibiot 48:261–266 10.7164/antibiotics.48.261 7730162

[CR59] Stadler M, Anke H, Sterner O (1995b) New metabolites with nematicidal and antimicrobial activities from the ascomycete *Lachnum papyraceum* (Karst.) Karst. IV. Structural elucidation of novel isocoumarin derivatives. J Antibiot 48:267–270 10.7164/antibiotics.48.267 7730163

[CR60] Stadler M, Anke H, Sterner O (1995c) Metabolites with nematicidal and antimicrobial activities from the ascomycete *Lachnum papyraceum* (Karst.) Karst. V. Production, isolation and biological activities of bromine-containing mycorrhizin and lachnumon derivatives and four additional new bioactive metabolites. J Antibiot 48:149–153 10.7164/antibiotics.48.149 7706125

[CR61] Stadler M, Anke H, Sterner O (1995d) New metabolites with nematicidal and antimicrobial activities from the ascomycete *Lachnum papyraceum* (Karst.) Karst. VII. Structure determination of brominated lachnumon and mycorrhizin a derivatives. J Antibiot 48:158–161 10.7164/antibiotics.48.158 7706127

[CR62] Stadler M, Anke H, Shan R, Sterner O (1995e) New metabolites with nematicidal and antimicrobial activities from the ascomycete *Lachnum papyraceum* (Karst.) Karst. VI. Structure determination of non-halogenated metabolites structurally related to mycorrhizin A. J Antibiot 48:154–157 10.7164/antibiotics.48.154 7706126

[CR63] Stadler M, Anke H, Dekermendijan K, Reiss R, Sterner O, Witt R (1995). New azaphilones from fruit bodies and mycelial cultures of the ascomycete *Bulgaria inquinans* Fr. Nat Prod Lett.

[CR64] Stadler M, Fouron J-Y, Sterner O, Anke H (1995). 1,2-Dihydroxymintlactone, a new nematicidal monoterpene isolated from the basidiomycete *Cheimonophyllum candidissimum* (Berk & Curt.) Sing. Z Naturforsch.

[CR65] Sterner O, Etzel W, Mayer A, Anke H (1997). Omphalotin, a new cyclic peptide with potent nematicidal activity from *Omphalotus olearius*. II. Isolation and structure determination. Nat Prod Lett.

[CR66] Takao K, Tsujita T, Hara M, Tadano K (2002). Asymmetric total syntheses of (+)-cheimonophyllon E and (+)-cheimonophyllal. J Org Chem.

[CR67] Thern B, Rudolph J, Jung G (2002). Total synthesis of the nematicidal cyclododecapeptide omphalotin A by using racemization-free triphosgene-mediated couplings in the solid phase. Angew Chem Int Ed Engl.

[CR68] Thorn RG, Barron GL (1984). Carnivorous mushrooms. Science.

[CR69] Thorn RG, Moncalvo J-M, Reddy CA, Vilgalys R (2000). Phylogenetic analyses and the distribution of nematophagy support a monophyletic *Pleurotaceae* within the polyphyletic pleurotoid-lentinoid fungi. Mycologia.

[CR70] Tsipouras A, Adefarati AA, Tkacz JS, Frazier EG, Rohrer SP, Birzin E, Rosegay A, Zink DL, Goetz MA, Singh SB, Schaeffer JM (1996). Ophiobolin M and analogues, noncompetitive inhibitors of ivermectin binding with nematocidal activity. Bioorg Med Chem.

[CR71] Ui H, Shiomi K, Yamaguchi Y, Masuma R, Nagamitsu T, Takano D, Sunazuka T, Namikoshi M, Ōmura S (2001). Nafuredin, a novel inhibitor of NADH-fumarate reductase, produced by *Aspergillus niger* FT-0554. J Antibiot.

[CR72] Welsh SJ, Williams RR, Birmingham A, Newman DJ, Kirkpatrick DL, Powis G (2003). The thioredoxin redox inhibitors 1-methylpropyl 2-imidazolyl disulfide and pleurotin inhibit hypoxia-induced factor 1α and vascular endothelial growth factor formation. Mol Cancer Ther.

[CR73] Yang Z-S, Li G-H, Zhao P-J, Zheng X, Luo S-L, Li L, Niu X-M, Zhang K-Q (2010). Nematicidal activity of *Trichoderma* spp. and isolation of an active compound. World J Microbiol Biotechnol.

[CR74] Yang Z, Yu Z, Lei L, Xia Z, Shao L, Zhang K, Li G (2012). Nematicidal effect of volatiles produced by *Trichoderma* sp. J Asia Pac Entomol.

[CR75] Yun C-H, Sugawara F, Strobel GA (1988). The phytotoxic ophiobolins produced by *Drechslera oryzae*, their structures and biological activity on rice. Plant Sci.

